# Exploring the Mechanisms of Electroacupuncture-Induced Analgesia through RNA Sequencing of the Periaqueductal Gray

**DOI:** 10.3390/ijms19010002

**Published:** 2017-12-25

**Authors:** Man-Li Hu, Hong-Mei Zhu, Qiu-Lin Zhang, Jing-Jing Liu, Yi Ding, Ju-Ming Zhong, Vitaly Vodyanoy, Ming-Xing Ding

**Affiliations:** 1 College of Veterinary Medicine, Huazhong Agricultural University, Wuhan 430070, China; humanli0727@163.com (M.-L.H.); han.dong.1988@163.com (H.-M.Z.); colynqiulin@163.com (Q.-L.Z.); Ljj779009242@163.com (J.-J.L.); dingyi@mail.hzau.edu.cn (Y.D.); 2 College of Physiology and Pharmacology, Auburn University, Auburn, AL 36849, USA; zhongju@auburn.edu (J.-M.Z.); vodyavi@auburn.edu (V.V.)

**Keywords:** transcriptome, electroacupuncture analgesia, RNA sequencing, the periaqueductal gray, goat

## Abstract

Electroacupuncture (EA) can relieve various pains. However, its mechanism in terms of the transcriptome is still not well-known. To explore the full profile of EA-induced molecular modification in the central nerve system, three twins of goats were selected for a match-paired experiment: EA stimulation (60 Hz, 30 min) and none-EA (control). Goats in the EA group showed an increased (*p* < 0.05) nociceptive threshold compared with the control goats. Experimental goats were sacrificed at 4 h of the experiment, and the periaqueductal grays were harvested for RNA sequencing. As a result, 2651 differentially expressed genes (1803 up-regulated and 848 down-regulated genes) were found and enriched in 30 Kyoto Encyclopedia of Genes and Genomes pathways and 149 gene ontology terms. EA-regulated five neuropeptide genes (*proenkephalin*, *proopiomelanocortin*, *preprodynorphin*, *diazepam-binding inhibitor* and *proprotein convertase 1 inhibitor*) were validated with quantitative PCR. Furthermore, up-regulated glutamate receptors, glutamate transporters, γ-aminobutyric acid (*GABA*) receptors, GABA transporters, synaptotagmins or mitogen-activated protein kinase (*MAPK*) genes might contribute to EA-induced analgesia through regulating the glutamatergic synapse, GABAergic synapse, MAPKs, ribosome or ubiquitin-proteasome pathways. Our findings reveal a full profile of molecular modification in response to EA and provide a solid experimental framework for exploring the mechanisms underlying EA-induced analgesia.

## 1. Introduction

Electroacupuncture (EA), an effective and quantifiable modern version of manual acupuncture, is widely used for relieving various pains [1,2,3]. Since 1960, numerous studies have explored central mechanisms underlying EA-induced analgesia (EAA). Han’s group infused cerebrospinal fluid from a rabbit receiving acupuncture into a naive rabbit and found the latter produced an analgesic effect [4], showing some mediators in the central nerve system (CNS) play a vital role in EAA. Early studies with classical physical, pharmacological and biochemical approaches demonstrated that some neurotransmitters (e.g., glutamate, γ-aminobutyric acid, acetylcholine, norepinephrine and serotonergic) take part in EAA [5]. After acupuncture-induced analgesic effect was found to be partly reversed by an opiate antagonist, naloxone [6], opioid peptide roles raised extensive concern. Later, β-endorphin, enkephalin, endomorphin, dynorphins and orphanin FQ in the CNS were confirmed to mediate EA-induced analgesic effect [7,8,9]. Besides, several non-opioid peptides, such as Cholecystokinin octapeptide [10,11,12], substance P [13,14,15] and angiotensin II [16], were verified to participate in EAA. Obviously, EAA should be a comprehensive result mediated by multiple substances, including neurotransmitters and neuromodulators in the CNS. So far, over 100 different neuropeptides are known to be released by different populations of neurons in the CNS [17]. However, whether most of these neuropeptides are involved in EAA is not clear. Neurons are basal units for CNS activities. Numerous intracellular molecules take part in neuronal regulation. Recently, studies indicate that EA down-regulates several intracellular molecules, such as c-Fos [18], p38 mitogen-activated protein kinase (MAPK) and extracellular signal-regulated kinase (ERK), in the spinal dorsal horn (SDH), and that the intrathecal injection of p38MAPK or ERK inhibitors enhances EAA [19,20]. Since neurons in different nuclei or areas vary in function and possess complex intracellular molecules, the roles of EA-mediated central molecules in analgesia modulation are largely unknown. In complete elucidation of EAA underlying mechanisms, high-throughput sequencing can be used as a powerful tool for exploring EA-induced functional changes by measuring thousands of gene expression profiles, and further bioinformatic analysis can be used to disclose various EA-related neuropeptide genes and pathway signal molecules.

Substantially, EA-induced analgesia is a manifestation of integrative processes at different levels of the CNS between afferent impulses from the nociceptive regions and impulses from the acupoints [9]. The periaqueductal gray (PAG) with the various neurons (e.g., opioidergic, serotonergic, GABAergic) [21,22,23,24] integrates ascending nociceptive or acupuncture signals from the SDH and the descending antinociceptive signals from amygdala, habenula nucleus and hypothalamus [25,26,27,28,29]. Then, integrated signals were delivered to the rostral ventromedial medulla, locus coeruleus and SDH for modulating central homeostasis and responding to nociceptive stimuli [30]. It has been verified that the PAG is a pivotal integrative center in acupuncture analgesia [9]. In order to thoroughly discover mechanisms of EAA, similar genetic background goats were assigned into an EA group and a control group in the present study and the PAG was taken at 4 h of the experimental treatment for RNA sequencing. Our results indicate that EAA-related neuropeptides, including *proenkephalin* (*PENK*), *proopiomelanocortin* (*POMC*), *preprodynorphin* (*PDYN*), *diazepam-binding inhibitor* (*DBI*) and *proprotein convertase 1 inhibitor* (*Pcsk1n*) as well as the glutamatergic synapse, GABAergic synapse, MAPKs, ribosome and ubiquitin-proteasome pathways, play significant roles in EAA. The aim of the present study was to explore the full profile of EA-induced molecular modification in the CNS. It was hypothesized that EA-induced analgesia involved various substances, especially neuropeptides, and signal pathways in the CNS. 

## 2. Results

### 2.1. Effects of Electroacupuncture (EA) on Nociceptive Threshold

**Figure 1:**
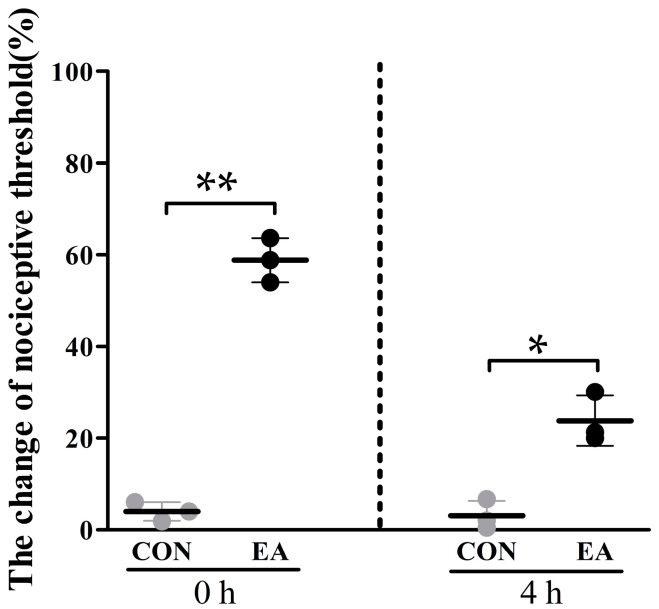
Analgesic effects induced by EA in goats (mean ± SD, %, *n* = 3). The nociceptive thresholds were measured with potassium iontophoresis. Nociceptive threshold increased at 0 and 4 h after EA stimulation. Goats in the control group were restrained as goats in EA group without needling and electricity. The significance of differences (** *p* < 0.01, * *p* < 0.05) was calculated by a *t*-test. The grey dots represent the control group. The black dots represent EA group.

EA analgesic effects were represented by the changes in nociceptive threshold (Figure 1). Compared with the control group, nociceptive threshold of EA group significantly increased (*p* < 0.05) by 58.80 ± 4.80% and 23.80 ± 5.49% at 0 and 4 h, respectively.

### 2.2. Global Analysis of Transcriptome

High-throughput sequencing was performed to analyze the expression profiles of goats’ PAG in EA and control groups. Six RNA sequencing libraries were unambiguously constructed. The metadata and raw sequences data files (six files) related to this experiment were deposited in the NCBI Sequence Read Archive (Bio project ID: PRJNA394953, accession numbers SRR5943641, SRR5943642, SRR5943643, SRR5943644, SRR5943645 and SRR5943646).

In total, 363.21 million raw sequence reads were generated. The Q30 percentage (proportion of nucleotides with quality value larger than 30 in reads) and GC contented proved that the sequence quality was good (Table 1 and Figure S1). After removing invalid reads, a total of 179.80 million and 172.51 million of clean bases were obtained from the control and EA group, respectively. The ratio of the matched reads of alignment ranged from 77.29% to 78.96% in the control group, and from 74.77% to 76.01% in EA group. In all matched reads, the uniquely mapped reads are more than 95.97%. The reads alignment to the goat genome indicated that the sequencing depth was sufficient for a differential expression analysis between the two groups of these goats.

### 2.3. Differentially Expressed Genes between EA and Control Group

**Figure 2:**
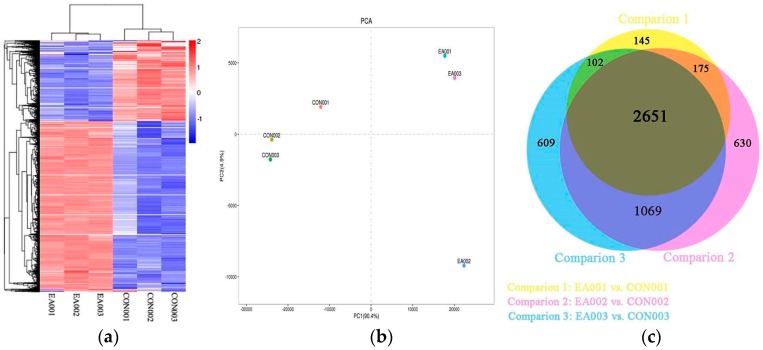
Overview of differentially expressed genes (DEGs). (**a**) Hierarchical clustering; (**b**) Principal component analysis; (**c**) An Venn diagram of DEGs. CON001, CON002 and CON003 were control groups. EA001, EA002 and EA003 were EA groups. Samples from same numbers were twin goats’ the periaqueductal gray.

To identify the differentially expressed genes between EA and the control group, the transcriptome data from PAGs of three pairs of goats were analyzed with DESeq. Expression levels were identified for significant differentially expressed genes (DEGs) by applying cutoff of *p*-value < 0.005 and log2 (Fold Change) > 1. Standardized hierarchical clustering analysis and Principal component analysis (PAC) of sample reads per kilobase of exon model per million mapped reads (RPKM) showed similar expression tendencies of samples within the control or EA groups (Figure 2a,b). The Venn diagram showed the overview of DEGs between samples (Figure 2c). There were 3073, 4431 and 4525 DEGs in each of the twin goats, respectively. A large proportion of DEGs among three twin goats were overlapped containing 2651 genes (1803 up-regulated and 848 down-regulated genes) in Table S1.

**Figure 3:**
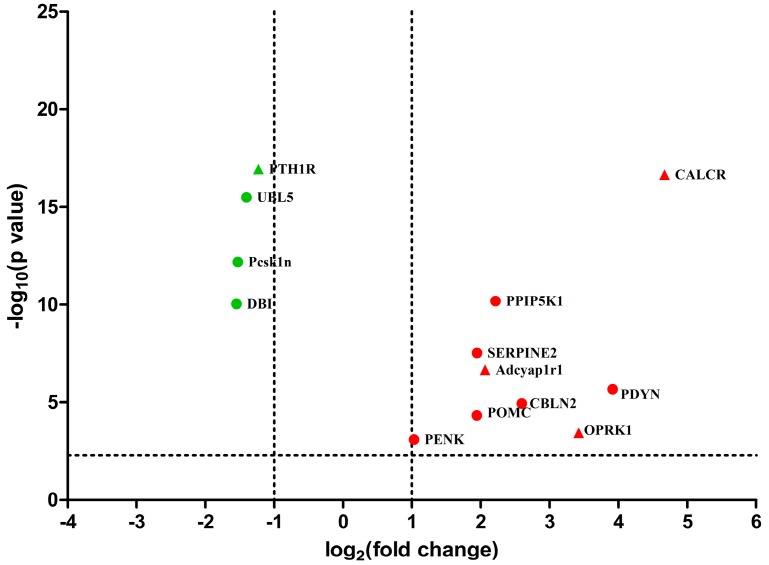
Volcano plot displaying differential expressed neuropeptide and neuropeptide receptor genes between EA and the control group. The *y*-axis corresponds to the value of log_10_ (*p* value), and the *x*-axis displays the log_2_ (fold change). The red dots represent the up-regulated neuropeptides genes (*p* < 0.005). The green dots represent the down-regulated neuropeptides genes (*p* < 0.005). The red triangles represent the up-regulated neuropeptide receptor genes (*p* < 0.005). The green triangles represent the down-regulated neuropeptide receptor genes (*p* < 0.005).

Based on database of neuropeptides [17], nine neuropeptide genes, including *PENK*, *POMC*, *PDYN*, *DBI*, *Pcsk1n*, *cerebellin-2* (*CBLN2*), *glia-derived nexin* (*SERPINE2*), *ubiquitin-like protein 5* (*UBL5*) and *inositol hexakisphosphate* and *diphosphoinositol-pentakisphosphate kinase 1* (*PPIP5K1*) as well as four neuropeptide receptor genes, including *opioid receptor kappa 1* (*OPRK1*), *calcitonin receptor* (*CALCR*), *pituitary adenylate cyclase-activating polypeptide type I receptor* (*Adcyap1r1*) and *parathyroid hormone/parathyroid hormone-related peptide receptor* (*PTH1R*) were found in DEGs (Figure 3 and Supplementary Table S1). Three opioid peptide precursors (*PENK*, *POMC* and *PDYN*) and one opioid peptide receptor (*OPRK1*) were up-regulated after EA stimulation. *Cerebellin-2*, *SERPINE2* and *PPIP5K1* were increased by 2.59, 1.94 and 2.21-fold, respectively, in EA group. Besides, the expression levels of *DBI*, *UBL5* and *Pcsk1n* were decreased after EA stimulation. *Calcitonin receptor* and *Adcyap1r1* were up-regulated while *PTH1R* was down-regulated.

### 2.4. Gene Ontology Overrepresentation Analysis

**Figure 4:**
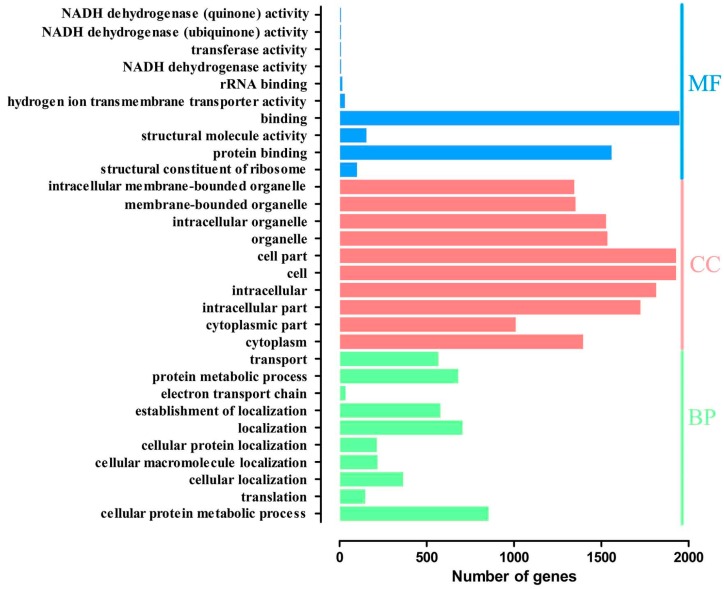
The most enriched gene ontology (GO) terms of differentially expressed genes between EA and the control group. Three aspects of Go terms are biological process (BP), cellular component (CC) and molecular function (MF).

The DEGs involved in enriched functional groups (*p* < 0.05) were 149 gene ontology (GO) terms (Table S2), containing 60 biological process terms (BP), 72 cellular component terms (CC), and 17 molecular function terms (MF). The ten most enriched categories of DEGs in the GO functional classification are shown in Figure 4. Among these GO terms, seven categories were connected to protein activities, including protein binding, structural constituent of ribosome, transport, protein metabolic process, cellular protein localization, translation and cellular protein metabolic process.

### 2.5. Kyoto Encyclopedia of Genes and Genomes Pathway Analysis

The DEGs were analyzed according to Kyoto Encyclopedia of Genes and Genomes (KEGG) functional annotations. Thirty enrichment pathways (*p* < 0.05) were presented (Table 2 and Table S2). The most enrich pathway was “ribosome”. The five enriched signal transduction pathways were MAPK signaling pathway, ErbB signaling pathway, HIF-1 signaling pathway, phosphatidylinositol signaling system and Wnt signaling pathway. In addition, neurodegenerative diseases and Substance dependence were also enriched, including Alzheimer’s disease, Parkinson’s disease, Huntington’s disease, amphetamine addiction, and morphine addiction. 

In the nervous system, seven pathways were identified from the 2651 DEGs in the KEGG analysis, including glutamatergic synapse (25 genes), GABAergic synapse (23 genes), dopaminergic synapse (30 genes), cholinergic synapse (22 genes), long-term potentiation (22 genes), neurotrophin signaling pathway (32 genes) and retrograde endocannabinoid signaling (26 genes) (Table 3 and Table S2). Except *Gng5*, *PPP1R1A*, *NGFRAP1*, *ATF4*, *FAAH*, *MATK*, *ATF4*, *ARHGDIG and MAP2K2*, all DEGs in these seven pathways were up-regulated after EA stimulation.

### 2.6. Differential Expression Genes Analysis and qPCR Validation

**Figure 5:**
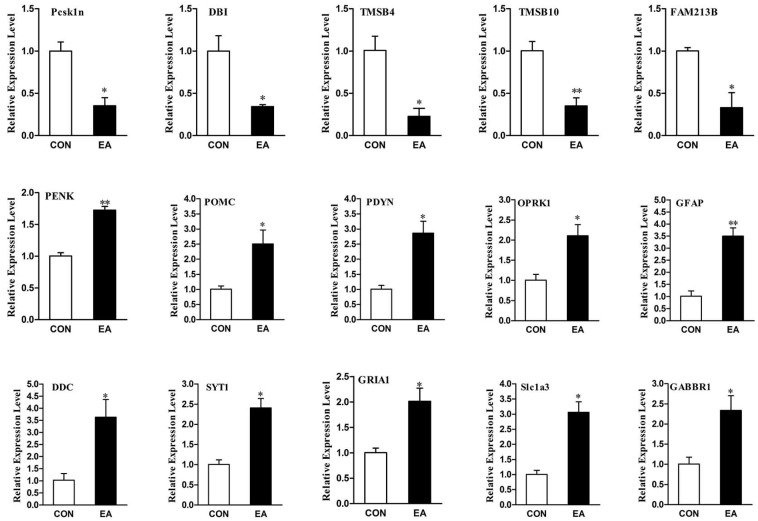
qRT-PCR validation of DEGs. The reliability of the sequencing data was validated by qRT-PCR. The change tendency of fifteen DEGs was consistent with the results from sequencing data. The data depicted by the *y*-axis were calculated using the expression values of 2^−ΔΔ*C*t^ and expressed as the mean ± SD in triplicate. The significance of differences in expression between samples was calculated by a t test. * Compared with the control *p* < 0.05. ** Compared with the control *p* < 0.01.

To validate the reliability of the sequencing data with the qRT-PCR, 15 genes (*Pcsk1n*, *DBI*, *PENK*, *POMC*, *PDYN*, *OPRK1*, *glutamate receptor 1*, *excitatory amino acid transporter 1*, *γ-aminobutyric acid type B receptor subunit 1*, *thymosin β-4*, *thymosin β-10*, *prostaglandin F synthase*, *Glial fibrillary acidic protein*, *Aromatic-L-amino-acid decarboxylase* and *Synaptotagmin-1*) were selected. As shown in Figure 5 and Table S3, all validated genes were consistent with the results from deep sequencing. Five genes, including *Pcsk1n*, *DBI*, *thymosin β-4* (*TMSB4*), *thymosin β-10* (*TMSB10*) and *prostaglandin F synthase* (*FAM213B*) were down-regulated (*p* < 0.05). Whereas, ten genes, including *PENK*, *POMC*, *PDYN*, *OPRK1*, *Glial fibrillary acidic protein* (*GFAP*), *Aromatic-l-amino-acid decarboxylase* (*DDC*), *Synaptotagmin-1* (*SYT1*), *glutamate receptor 1* (*GRIA1)*, *excitatory amino acid transporter 1* (*Slc1a3*) and *γ-aminobutyric acid type B receptor subunit 1* (*GABBR1)* were up-regulated (*p* < 0.05).

## 3. Discussion

Proper species is important factors influencing EA analgesic effect. Clinical studies have shown that EA in combination with analgesics reduces the required dose of analgesics in varies species. The potent analgesia induced by EA in goats (75% reduction of analgesic) [31,32] was superior than that in humans (48% reduction of analgesic) [33] or rats (50% reduction of analgesic) [34] if the species were not considered. Obviously, goats are optimal animals for studying the mechanism underlying EA-induced analgesia. Acupuncture frequencies and points are other important factors. Liu et al. [32] used 36 Hz to stimulate the set of Baihui, Santai, Ergen and Sanyangluo points in goats and obtained good analgesic effects. Cheng et al. [35] performed different frequencies (2, 40, 60, 80 and 100 Hz) to stimulate the same set of acupoints in goats, and found that 60 Hz induced more potent analgesia than any of the other frequencies. In the present study, EA at 60 Hz was used to stimulate a set of Baihui, Santai, Ergen, and Sanyangluo acupoints in goats, which increased the nociceptive threshold by 58.80 ± 4.80% after EA stimulation, which has a similar tendency to previous studies [32,35,36].

EA has been widely used for alleviating diverse pains under morbid conditions [37,38]. Since acupuncture analgesia is a manifestation of complex processes involving diverse substances in the CNS, the central mechanism by which EA modifies nociception is still a conundrum. Effect of EA on physiological pain responses is a basis for understanding EA-induced analgesia. Ke et al. [39] explored some probable EAA-related DEGs in rat PAGs and spinal cords under the physiological condition with cDNA microarray hybridization. However, this method with cDNA microarray hybridization and pooled RNAs only discovered limited information compared with high throughput screening and RNAs from individual samples. In addition, rats might not provide an optimized EAA-related gene expression response because they display analgesic effects inferior to goats and different responses to EA (non-responders and responders) [40]. In the present study, Illumina deep sequencing was used to obtain transcriptome data from three purebred twins of goats with almost the same genetic background to explore EAA-related DEGs and its central regulatory mechanisms.

Several researches indicated that EA-induced analgesia lasted for 12 h [41] while EA-induced mRNA expression began in 0–3 h and reached a peak level during 2–24 h in rats [42,43,44]. It is proteins (peptides), rather than their genes, that have direct effect on EAA. Cheng et al. [36] found that the nociceptive threshold reached the first and second peak at 0 and 8 h, respectively, while the mRNA levels of endogenous opioid peptides (*PENK*, *POMC* and *PDYN*) and opioid receptors (*OPRK1*, *opioid receptor mu 1* and *opioid receptor delta 1*) increased at 0 h and reached the peak during 4–6 h after EA stimulation in goat PAGs, which suggested that the increased expression level of mRNA at 4 h might contribute to the second peak formation of the EA-induced nociceptive threshold at 8 h. Therefore, in the present study, PAG samples were taken from naïve goats at 4 h after EA stimulation for RNA sequencing. Several studies indicated that no changes in both nociceptive threshold and c-fos expression (a activating marker in the CNS) were found between the control group and the sham group (needling without electricity) in rats [45,46]. Similarly, the mRNA and protein levels of EAA-related substances including opioid peptides, c-fos and c-jun in control goats were not different from those in the sham goats [35,47,48]. Therefore, the control group was used instead of the sham group in our present study. The correlation analysis and cluster analysis (Figure S2 and Figure 2) of the transcriptome showed high biological repeatability and less individual difference in PAG samples. To decrease the influence of individual difference in genetic background, DEGs were selected from each twin of goats. DEGs within each twin of goats were compared further, and 2651 common DEGs among three twins of goats were selected for further analysis (NCBI Sequence Read Archive; Bio Project ID: PRJNA 394953).

It has been verified that EA exerts its analgesic effect through the release of endogenous opioid peptides [9,15,49]. EA could increase both protein and gene expressions of endogenous opioid peptides and opioid peptide receptors in the CNS [9,36,50]. However, the microinjection of opioid receptor antagonists in the CNS partly reversed EA-induced analgesic effect [51,52,53], which suggests that besides endogenous opioid peptides, other substances may be involved in EAA in the present study. We found three opioid peptide precursors (*PENK*, *POMC* and *PDYN*) and opioid peptide receptor (*OPRK1*) were up-regulated after EA stimulation and consistent with previous studies [36,54]. Interestingly, *Pcsk1n* and *DBI* neuropeptides were also found to be involved in EAA (Figure 3). Pcsk1n is a specific endogenous inhibitor of *proprotein convertase 1* (Pcsk1), which may inhibit Pcsk1-mediated formation of *POMC* and *PENK* [55,56]. The down-regulation of *Pcsk1n* might dismantle the inhibition of Pcsk1, contribute to the formation of POMC and PENK, and then enhance EAA. DBI, a 10 kDa endogenous polypeptide, is widely distributed in the CNS [57]. It has ability to inhibit diazepam binding to γ-aminobutyric acid type A (GABAa) receptor [58]. Pharmacological studies found that the intrathecal administration of a GABAa receptor antagonist suppressed acupuncture analgesia [59], whereas the intrathecal administration of GABA receptor agonist diazepam potentiated EAA [60]. Therefore, the down-regulation of *DBI* probably dismantles the inhibition of diazepam binding to the GABAa receptor, and contributes to EAA. Besides neuropeptides mentioned above, lots of other peptides’ genes significantly changed in the present study (Table S1). For instance, *TMSB4* was down-regulated after EA stimulation and verified with qRT-PCR (Figure 5). The protein thymosin β4 (Tβ4), a 44-amino acid pleiotropic polypeptide, has neuroprotective effect [61] and could promote brain development [62,63]. Several studies reported that Tβ4 participated in p38MAPK [64] and PI3K [65] signal pathways. Since these are also related to EA-induced anti-hyperalgesia [66,67,68], the relationships between Tβ4 and EA under both physiological and pathological conditions are worthy being further investigated.

Glutamatergic synapse and GABAergic synapse pathways were found in KEGG analysis of DEGs. It is well-documented that glutamate and γ-aminobutyric acid (GABA) play a pivotal role in the spinal transmission of nociceptive information and the central sensitization, and that their receptors have been confirmed to regulate EAA. Previous studies showed that EA could antagonize CFA- or nerve injury-induced increase in spinal glutamate receptors (NR1, NR2 and GluR1), and result in the relief of hyperalgesia [69,70,71,72]. Furthermore, pharmacological studies showed that EA in combination with a *N*-Methyl-d-aspartate (NMDA) receptor antagonized carrageenan- and CFA-induced hyperalgesia and neuropathic hyperalgesia [70,73]. These studies suggest EA can induce anti-hyperalgesic effects through its inhibition of spinal NMDA receptors. However, EA could increase spinal NR1/NR2B and hippocampal GluR1 in normal rats, and GluR1 of amygdala in chronic neuropathy hyperalgesia rats, which suggests that roles of glutamate receptors are complicated and vary in different areas of EAA-involved CNS. GABA receptors seem to contribute to EAA. The intracerebroventricular administration of γ-aminobutyric acid type B (GABAb) receptor antagonists clearly decreased acupuncture analgesia [74]. Moreover, the intrathecal administration of GABAa or GABAb receptor antagonists partially blocked acupuncture analgesia [59,75,76]. Evidence has shown that glutamate transporters are also involved in EA analgesia. Cui et al. [42] demonstrated that spinal glutamate transporters (GLAST, GLT-1 and EAAC1) were up-regulated by EA in healthy rats with nociceptive threshold increased. Further, pharmacological studies showed that CFA- [77] and spared nerve injury-induced [78] decline in spinal GLAST and GLT-1 could be reversed by EA stimulation, showing they contributed to EA-induced antinociceptive effect in rats. In the present study, glutamate receptors (*GRIA1/GluR1*, *GRIA2/GluR2*, *GRIA3* and *GRIN1/NR1*), glutamate transporters (*SLC1A1/EAAC1* and *SLC1A3/GLAST*), GABA receptors (*GABRA1*, *GABRA3*, *GABRA5*, *GABBR1* and *GABBR2*), GABA transporter 1 (*SLC6A1*), vesicular glutamate transporter 2 (*SLC17A6*), vesicular inhibitory amino acid transporter (*Slc32a1*) and sodium-coupled neutral amino acid transporter (*SLC38A2*) being enriched in Glutamatergic synapse and/or GABAergic synapse pathways were all up-regulated in the PAGs of goats after EA stimulation. The roles of these glutamate receptors and GABA receptors and their transporters in EAA are worth further investigated.

Besides glutamatergic synapse and GABAergic synapse pathways, other two synapse-related pathways, Dopaminergic synapse and Cholinergic synapse were also enriched. It has been verified that glutamate, GABA, acetylcholine and dopamine as neurotransmitters take part in EAA [5,9]. Previous studies indicated that the Ca^2+^-binding synaptic-vesicle protein-SYT-1 contributed to the synaptic vesicle fusion process of neurotransmitter release [79,80]. In the present study, synaptotagmins (*SYT1*, *SYT4*, *SYT11* and *SYT13*) were up-regulated, which suggested they may contribute to EA-induced release of neurotransmitters. Five signal transduction pathways, MAPK signaling pathway, Wnt signaling pathway, HIF-1 signaling pathway, phosphatidylinositol signaling system and ErbB signaling pathway were enriched in the present study. MAPKs are important signal pathways in regulation of nociceptive sensitivity [81,82,83,84]. Increasing evidence has shown inflammatory and neuropathic hyperalgesia-induced increasing expression of spinal MAPKs (e.g., p38MAPK, JNK and ERK) [66,67,85,86,87] could be reversed by EA stimulation resulting in relief of hyperalgesia [66,67,88]. In the present study, *JNK3/MAPK10*, *ERK2/MAPK1*, *ERK3/MAPK6* and *ERK4/MAPK4* were up-regulated in the PAG, which are inconsistent with the changes of these genes in the spinal cord under pathological conditions. EA possesses favorably bidirectional regulation for some intracellular molecules in the CNS; for example, EA increases c-Fos expression in the physiological condition [47,48,89], but reverses pathological hyperalgesia-induced increase in spinal c-Fos [18,90]. In the PAG, both facilitatory and inhibitory nociceptive modulatory systems are believed to participate in EAA [5,9,91]. The molecular mechanisms underlying EAA remain much unknown. Several studies demonstrated that EA has ability to cure cerebral injury through up-regulating HIF-1α protein [92,93], wnt l and β-catenin [94,95] in the CNS. Since there are no studies investigating the roles of Wnt signaling pathway, HIF-1 signaling pathway, phosphatidylinositol signaling system and ErbB signaling pathway in EAA, roles of these four signal transduction pathways participating in EAA need to be confirmed in further studies.

We performed GO overrepresentation analysis of DEGs and found that seven categories were connected to protein activities including protein binding, structural constituent of ribosome, transport, protein metabolic process, cellular protein localization, translation and cellular protein metabolic process. Furthermore, DEGs were enriched in ribosome and proteasome involved in protein metabolism with KEGG pathway analysis. These enriched GO terms and KEGG pathways suggested that EA could widely influence protein activities which might be a new mechanism of EAA. In the present study, most ribosome protein genes were down-regulated by EA, showing EA decreased the corresponding ribosome proteins, and thus reduce protein synthesis. In addition, the ubiquitin-proteasome system is responsible for protein degradation [96,97]. E3 ubiquitin-protein ligase genes were highly expressed and proteasome genes were less expressed after EA administration in current study, showing EA might influence protein degradation. Kim et al. [77] found that both EA and MG-132 (a proteasome inhibitor) antagonized CFA-induced down-regulation of spinal GLAST and GLT-1, suggesting that EA probably mediates GTs regulation through the ubiquitin-proteasome pathway in inflammation hyperalgesia. These results indicated that EA might regulate other EAA-related signal molecules through regulating protein metabolism (synthesis and degradation), which is worth being further explored.

There are several limits which may exist in the present study. The sample size satisfied the deep sequencing, but may not be enough for the statistical analyses of nociceptive thresholds. Despite the small sample size, EA potent treatment effects, being consistent with a lot of previous reports with 6–8 sample numbers [31,36,48], made the experimental power more than 90%. In addition, we can only discuss several DEGs and enriched pathways that could be supported by the literature because of limited information about EAA in the available online data. The EA-induced neuropeptides, intracellular molecules and signaling pathways found in our study need to be further investigated to fully elucidate the central EAA mechanism.

## 4. Materials and Methods

### 4.1. Animal Preparation

Three twins of Boer goats (pedigree, female, one-year-old, weighing 28–32 kg) from the same father were purchased from the breeding goats farm of Hubei Province. The goats were housed in a same management condition with food and water ad libitum. Quiet environment and the room temperature 22 ± 2 °C were maintained during experiment. The goats were accustomed to being approached and adapted to the ambient environment and prostrate restraint (1 h/day) for two weeks. Three goats of EA group were randomly selected from three twins of goats and marked as EA001, EA002 and EA003. The rest three goats were assigned to the control group and marked as CON001, CON002 and CON003. Goats marked same number were twins. All the experimental methodology and research on animals were strictly performed according to the guidelines established by the Ministry of Science and Technology for experimental animals. The experimental protocol was approved by the Animal Care Center, College of Veterinary Medicine, Huazhong Agricultural University, Wuhan, China (Permission number: HZAUGO-2016-006, Permission data: 2016-06-07).

### 4.2. EA Application

**Figure 6:**
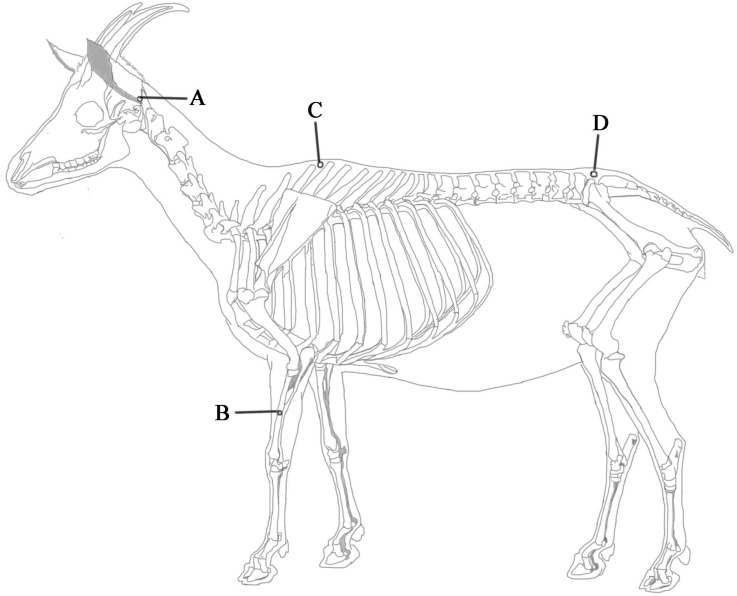
The location of acupoints in goat. (**A**) Ergen; (**B**) Sanyangluo; (**C**) Santai; (**D**) Baihui.

For EA stimulation, the set of Baihui, Santai, Ergen and Sanyangluo acupoints was selected. Here the acupoints were nominated with Pinyin Naming System instead of the Meridian Numbering System because animal’s meridians were not completely recorded. The anatomical locations of these acupoints have been described in detail for veterinary medicine [32,48] and shown in Figure 6. The Baihui acupoint was identified on the dorsal midline between the spinous processes of the last lumbar and the first sacral vertebrae. The Santai acupoint was identified on the dorsal midline between the spinous processes of the fourth and fifth thoracic vertebrae. The Ergen acupoints were identified bilaterally, with each at the pit ventrocaudal to the ear base between the ear base and the cranial border of the transverse process of the atlas on each side. The Sanyangluo acupoints were also identified bilaterally, with each at approximately 5 cm ventral to the lateral tuberosity of the radius in the groove between the common digital extensor and lateral digital extensor muscles of the forelimb. The Ergen and Sanyangluo acupoints on the left side of the body were chosen in this study. Needle insertion and EA were conducted with the method according to previous studies [35]. A pair of wires from one output of the WQ-6F Electronic Acupunctoscope (BeijingXindonghua Electronic Instrument Co., Ltd., Beijing, China) connected needles at Baihui and Santai acupoints. Other pair of wires from the same machine connected needles at Ergen and Sanyangluo acupoints. The frequency and voltage of stimulation were set to 60 Hz and 3.2 V, respectively [36,48]. The goats of EA group were administered EA for 30 min. The goats of control group were restrained in the same manner as goats of EA group without needling and electricity.

### 4.3. Determination of Nociceptive Threshold.

Nociceptive threshold was assessed just before (−0.5 h) and 0 and 4 h after EA administration by one skilled person who was blinded to the goat assignments. Nociceptive threshold was measured with potassium iontophoresis by a direct current induction therapy apparatus (Shantou Medical Equipment Factory Co., Ltd., Shantou, China). The site was shaved and cleaned with soap and water and sterilized with 75% ethanol. Two electrodes were soaked with saturated potassium chloride and placed 1–2 cm apart on the left flank skin. The pulse direct current delivered potassium ions into the subcutaneous tissues while voltage was continuously increased. When obvious contraction of the local skin and muscle along with head turning toward the abdomen, back hunching, and body eluding movement were observed, the voltage level was recorded. The procedure was repeated three times with 5 min interval. Mean voltages before and after EA were used to calculate the change percentage of nociceptive threshold.

### 4.4. Extraction of RNA and Sequencing Using RNA-Seq Technology

**Figure 7:**
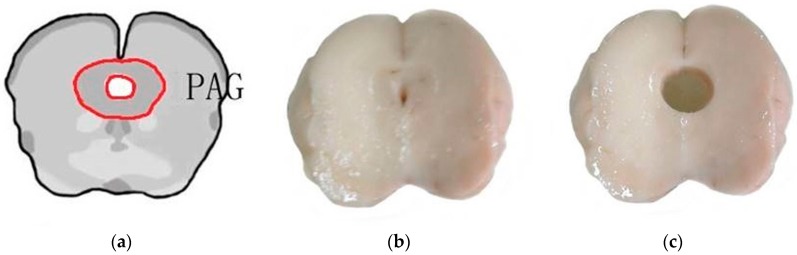
The location of the periaqueductal gray (PAG). (**a**) The sketch of the PAG location; (**b**) The brain section containing the PAG; (**c**) The brain section after sampling.

Differential expression genes in the PAGs of goats were conducted by RNA sequencing. Because there was no difference in nociceptive threshold between the control group and the sham group (needling without electricity) in previous studies [35,47,48], the goats that were used for the differential genes expression analysis were treated with EA (60 Hz, 30 min) or non-EA (control). Six goats were deeply anesthetized with intravenous administration of xylidinothiazoline (3 mg/kg) at 4 h after experimental treatment. The brain was taken out of the skull and placed on a DEPC water-treated plate with the ventral surface up. Then it was transected into three sections through the sulcus between cerebral peduncles and pons and the sulcus between pons and medulla oblongata, respectively, with the method described by Cheng et al. [36]. The midbrain was taken and evenly cut into three subsections. The PAG was obtained with 6 mm diameter plastic tubes dealt with 1% DEPC solution from the middle subsection from midbrain and then put into liquid nitrogen. The location of the PAG was identified according to the photographic atlas of the goat brain and the morphological characteristics of the neurons [98] in Figure 7. 

Six PAG tissues from EA and the control goats were obtained. Experimental total RNA of the PAG for sequencing and qPCR analysis was extracted using the Trizol reagent (Invitrogen, Carlsbad, CA, USA) according to the manufacturer’s protocol. RNA quality was assessed with a Lab-on-chip Bioanalyzer 2100 (Agilent Technologies, Palo Alto, CA, USA). The purity, concentration and integrity of RNA were assessed by NanoPhotometer^®^ spectrophotometer (Thermo Fisher Scientific, Waltham, MA, USA), Qubit^®^ RNA Assay Kit in Qubit^®^ 2.0 Flurometer (Life Technologies, Pleasanton, CA, USA) and RNA Nano 6000 Assay Kit of the Agilent Bioanalyzer 2100 system (Agilent Technologies), respectively. Transcriptome sequencing was performed with Novogene Bioinformatics Technology Co., Ltd., Beijing, China (www.novogene.cn). Six complementary DNA libraries were constructed using NEBNext mRNA Library PrepReagent Set for Illumina (Illumina, Santiago, CA, USA) following the manufacturer’s protocol. The libraries were sequenced on an Illumina Hiseq 2000 platform and 100 bp paired-end reads were generated.

### 4.5. Quality Control for Raw Sequencing Data

Raw data (raw reads) of fastq format were firstly processed through in-house Perl scripts. In this step, clean data (clean reads) were obtained by removing reads containing adapter, reads containing poly-N and low-quality reads from raw data. Then, Q30 and GC-content of the clean data were calculated. All the downstream analyses were based on the clean data with high quality. 

### 4.6. Transcriptome Analysis for mRNA Data

Paired-end clean reads were mapped to the Capra hircus reference genome with TopHat-2 [99]. The reads numbers mapped to each gene were counted with HTSeq [100]. And then RPKM of each gene was calculated based on the length of the gene and reads count mapped to this gene [101]. Pearson correlation between samples was tested to verify reliability and rationality of the design in biological replicates. In addition, a standardized hierarchical clustering analysis and PAC of RPKM was used to get an overview of gene expression difference between samples. The standardized hierarchical clustering analysis was processed using R package with Euclidean and Ward, respectively. Principal component analysis was processed with Omicshare tools in website http://www.omicshare.com/.

Prior to differential gene expression analysis for each sequenced library (without biological replicates), the read counts were adjusted by edgeR program package through one scaling normalized factor [102]. Differential expression analysis of the PAG within each twin goats was performed using the DEGSeq R package (1.20.0) [103]. The *p* values were adjusted using the Benjamini & Hochberg method [104]. Corrected *p*-value of 0.005 and log2 (Fold change) of 1 were set as the threshold for significantly differential expression. DEGs within each twin goats were further compared, and common DEGs among three twins of goats were selected for further analysis. Differential expression analysis of EA and control groups (three biological replicates per groups) was performed using the DESeq R package [105]. Venn diagram was used to perform DEGs within each twin goats and common DEGs in three twin goats.

The common DEGs in three twin goats were blast to neuropeptides database (http://www.neuropeptides.nl/tabel%20neuropeptides%20linked.htm) [17] with blastx tool. E_value of 1 × 10^−5^ was set as the threshold.

Functional annotations of all assembled unigenes were conducted by searching against the following databases: Swiss-Prot protein (Swiss-Prot), KEGG Ortholog database (KO), and Gene Ontology database (GO). Gene Ontology (GO) overrepresentation analysis of DEGs was implemented by the GOseq R package [106] in which *p*-value based on a Poisson exact test. GO terms with corrected *p* value less than 0.05 were considered significantly enriched by DEGs. 

KOBAS software [107] was used to implement KEGG pathways overrepresentation analysis of DEGs and test the statistical enrichment. KEGG pathways with corrected *p* value less than 0.05 were considered significantly enriched by DEGs.

### 4.7. Validation of Differential Expression Genes

The RNA sequencing data were confirmed through qRT-PCR. Ten up-regulated and five down-regulated DEGs of interest were selected for qRT-PCR validation. These genes almost covered the entire spectrum of differential expression changes (log2 Fold change. from −1.3 to +5) as well as the absolute expression levels (rpkm of DEGs from 0.1 to 10,468). A list of primers of selected genes was shown in Table S3. Goat β-actin gene was used as an internal control gene for normalization of cDNA loading differences in this experiment. Eight hundred nanograms of total RNA from each sample were reverse-transcribed into cDNA using the First Strand cDNA Synthesis Kit ReverTra Ace-α (ToYobo Co., Ltd., Osaka, Japan). SYBR^®^ Premix Ex TaqTMII (TaKaRa Co., Ltd., Dalian, China) were used to perform qRT-PCR with standard protocols on the Step One Real-Time PCR System (Applied Biosystems, Foster City, CA, USA). The 2^−ΔΔ*C*t^ method was used to analyze expression levels. Data were expressed as the mean ± SD from triplicate determinations.

### 4.8. Statistical Analysis.

The statistical analyses of the nociceptive thresholds differences between EA and control group were tested by Student’s *t*-test in each time point. Comparisons between qPCR datasets were calculated using Student’s *t*-test. All data and tests were analyzed with SPSS 21.0 (SPSS Inc., Chicago, IL, USA). *p* < 0.05 was considered statistically significant.

## 5. Conclusions

Experimental goats between EA and control groups displayed a distinct difference in nociceptive threshold and molecular modifications. Goats in the EA group showed increased pain threshold compared with the control goats. We performed RNA sequencing to identify the full profile of EA-induced molecular modification in goat PAG. Our results indicate that five neuropeptides genes (*PENK*, *POMC*, *PDYN*, *DBI* and *Pcsk1n*) as well as glutamatergic synapse, GABAergic synapse, MAPK, ribosome and ubiquitin-proteasome pathways might contribute to EA-induced analgesia. The results generated by this study have provided information, not only on responsible genes and molecular pathways, but also on possible mechanisms involving the process of EA-induced analgesia. Further pathological and physiological studies of these candidate genes and pathways are warranted in the future.

## Figures and Tables

**Table 1:** The summary of the RNA sequencing analysis data.

Parameters	Control Group			Electroacupuncture (EA) Group		
	CON001	CON002	CON003	EA001	EA002	EA003
Raw reads	63566836	63131918	58793220	60857264	57505218	59363680
Clean reads (A)	61436432	61325678	57038940	59053276	55818042	57636846
Error rate (%)	0.003	0.003	0.003	0.003	0.003	0.003
Q30 (%)	92.43	92.32	92.67	92.02	91.84	92.28
GC content (%)	48.07	49.28	48.03	48.56	49.20	49.07
Total mapped (B)	47483936	48420282	44162522	44152353	42426278	43177715
Multiple mapped	1824973	1827216	1778697	1357349	1654599	1290277
Uniquely mapped (C)	45658963	46593066	42383825	42795004	40771679	41887438
Ratio of (B) and (A)	77.29%	78.96%	77.43%	74.77%	76.01%	74.91%
Ratio of (C) and (B)	96.16%	96.23%	95.97%	96.92%	96.10%	97.02%

**Table 2:** Enriched pathways of differentially expressed genes (DEGs).

Pathway	Pathway Id	Count	*p*-Value
Ribosome	bta03010	73	0
Oxidative phosphorylation	bta00190	76	7.86 × 10^−14^
Alzheimers’ disease	bta05010	82	6.12 × 10^−10^
Parkinson’s disease	bta05012	67	2.14 × 10^−9^
Huntington’s disease	bta05016	83	1.29 × 10^−8^
Citrate cycle (TCA cycle)	bta00020	19	1.58 × 10^−5^
Endocytosis	bta04144	76	2.66 × 10^−5^
ErbB signaling pathway	bta04012	31	0.000163
Long-term potentiation	bta04720	22	0.00085
Phosphatidylinositol signaling system	bta04070	28	0.000888
Proteasome	bta03050	18	0.001086
GABAergic synapse	bta04727	23	0.001395
Inositol phosphate metabolism	bta00562	26	0.001604
Bacterial invasion of epithelial cells	bta05100	24	0.001823
Adherens junction	bta04520	27	0.002634
Fc gamma R-mediated phagocytosis	bta04666	34	0.003565
HIF-1 signaling pathway	bta04066	38	0.004882
Retrograde endocannabinoid signaling	bta04723	26	0.004904
Tight junction	bta04530	44	0.005358
Dopaminergic synapse	bta04728	30	0.007337
Glutamatergic synapse	bta04724	25	0.009755
Protein processing in endoplasmic reticulum	bta04141	54	0.016489
Amphetamine addiction	bta05031	25	0.017283
Renal cell carcinoma	bta05211	25	0.017283
Cholinergic synapse	bta04725	22	0.020068
Gap junction	bta04540	29	0.040443
Neurotrophin signaling pathway	bta04722	32	0.041074
Wnt signaling pathway	bta04310	47	0.04123
Morphine addiction	bta05032	30	0.043044
MAPK signaling pathway	bta04010	76	0.04338

**Table 3:** DEGs in pathways of the nervous system.

Pathways in KEGG	Genes
Glutamatergic synapse	*GNAO1*, *GNB1*, *GNAI1*, *GNAI2*, *PRKACA*, *PRKCB*, *ADCY7*, *ADCY2*, *ITPR1*, *PLCB4*, *SLC38A2*, *GLUL*, *GRIA1*, *GRIA2*, *GRIA3*, *PPP3CB*, *PPP3CA*, *Slc1a3*, *SLC1A1*, *SLC17A6*, *GRIN1*, *ADRBK1*, *Ppp3r1*, *MAPK1*, *CHP1*
GABAergic synapse	*GNAO1*, *GNB1*, *GNAI1*, *GNAI2*, *PRKACA*, *PRKCB*, *ADCY7*, *ADCY2*, *SLC38A2*, *GLUL*, *GABRA1*, *GABRA3*, *GABRA5*, *GABBR1*, *GABBR2*, *GABRG2*, *GABARAPL1*, *SLC6A1*, *Src*, *Slc32a1*, *GAD1*, *NSF*, *PLCL1*
Dopaminergic synapse	*GNAO1*, *GNB1*, *GNAI1*, *GNAI2*, *PRKACA*, *PRKCB*, *AKT2*, *AKT3*, *Camk2g*, *Camk2d*, *CAMK2B*, *CAMK2A*, *ATF4*, *ITPR1*, *PLCB4*, *GRIA1*, *GRIA2*, *GRIA3*, *PPP3CB*, *PPP3CA*, *Ppp1cb*, *Ppp2r5a*, *PPP2R2C*, *PPP2CA*, *SCN1A*, *Mapk10*, *DDC*, *KIF5B*, *Kif5a*, *MAOB*
Cholinergic synapse	*GNAO1*, *GNB1*, *GNAI1*, *GNAI2*, *PRKACA*, *PRKCB*, *AKT2*, *AKT3*, *Camk2g*, *Camk2d*, *CAMK2B*, *CAMK2A*, *ATF4*, *ADCY7*, *ADCY2*, *ITPR1*, *PLCB4*, *PIK3R2*, *PIK3CB*, *PIK3R1*, *JAK2*, *GNA11*
Long-term potentiation	*Ppp1cb*, *PPP3CA*, *GRIA2*, *RAP1B*, *PRKACA*, *Camk2g*, *CAMK2A*, *ITPR1*, *GRIA1*, *RPS6KA6*, *CHP1*, *Ppp3r1*, *MAPK1*, *CAMK2B*, *PLCB4*, *PPP3CB*, *Camk2d*, *GRIN1*, *MAP2K2*, *ATF4*, *PRKCB*, *PPP1R1A*
Neurotrophin signaling pathway	*CAMK2B*, *PRDM4*, *RAP1B*, *PIK3CB*, *Camk2g*, *Crk*, *CAMK2A*, *RPS6KA6*, *GAB1*, *MAPK1*, *SHC1*, *Camk2d*, *Mapk10*, *PIK3R2*, *Map2k7*, *AKT3*, *SOS2*, *Map3k5*, *YWHAE*, *PTPN11*, *RAPGEF1*, *RPS6KA5*, *PLCG1*, *PIK3R1*, *AKT2*, *NFKB1*, *ABL1*, *MAP2K2*, *ARHGD*, *ATF4*, *MATK*, *NGFRAP1*
Retrograde endocannabinoid signaling	*Gng5*, *FAAH*, *ADCY2*, *GRIA3*, *GNB1*, *ABHD6*, *GNAI2*, *Slc32a1*, *Mapk10*, *PRKCB*, *PLCB4*, *MAPK1*, *MGLL*, *GNAO1*, *ADCY7*, *GRIA1*, *ITPR1*, *PRKACA*, *RIMS1*, *GRIA2*, *GABRA3*, *GABRA1*, *SLC17A6*, *GABRA5*, *GNAI1*, *GABRG2*

## Supplementary Materials

Supplementary materials can be found at www.mdpi.com/1422-0067/19/1/2/s1.

## Abbreviations

EA	electroacupuncture
EAA	EA-induced analgesia
CNS	central nerve system
MAPK	mitogen-activated protein kinase
ERK	extracellular signal-regulated kinase
PAG	periaqueductal gray
PCA	principal component analysis
SDH	spinal dorsal horn
PENK	proenkephalin
POMC	proopiomelanocortin
PDYN	preprodynorphin
DBI	diazepam-binding inhibitor
Pcsk1n	proprotein convertase 1 inhibitor
RPKM	Reads Per Kilobase of exon model per Million mapped reads
DEGs	differentially expressed genes
OPRK1	opioid receptor kappa 1
GO	gene ontology
KEGG	kyoto Encyclopedia of Genes and Genomes
GRIA1	glutamate receptor 1
Slc1a3	excitatory amino acid transporter
SYT1	synaptotagmin-1
GABBR1	γ-aminobutyric acid type B receptor subunit 1
GABA	γ-aminobutyric acid
GABAa	γ-aminobutyric acid type A
GABAb	γ-aminobutyric acid type B
TMSB4	thymosin β4 (gene)
Tβ4	thymosin β4 (protein)
NMDA	*N*-Methyl-d-aspartate
