# Supplementary figures and images for: Exploring the Mechanisms of Electroacupuncture-Induced Analgesia through RNA Sequencing of the Periaqueductal Gray
Source: Int J Mol Sci. 2017 Dec 25;19(1):2. doi: 10.3390/ijms19010002 (PMC5795954; doi:10.3390/ijms19010002)

Supplementary figure 1: Fast QC (quality control) data

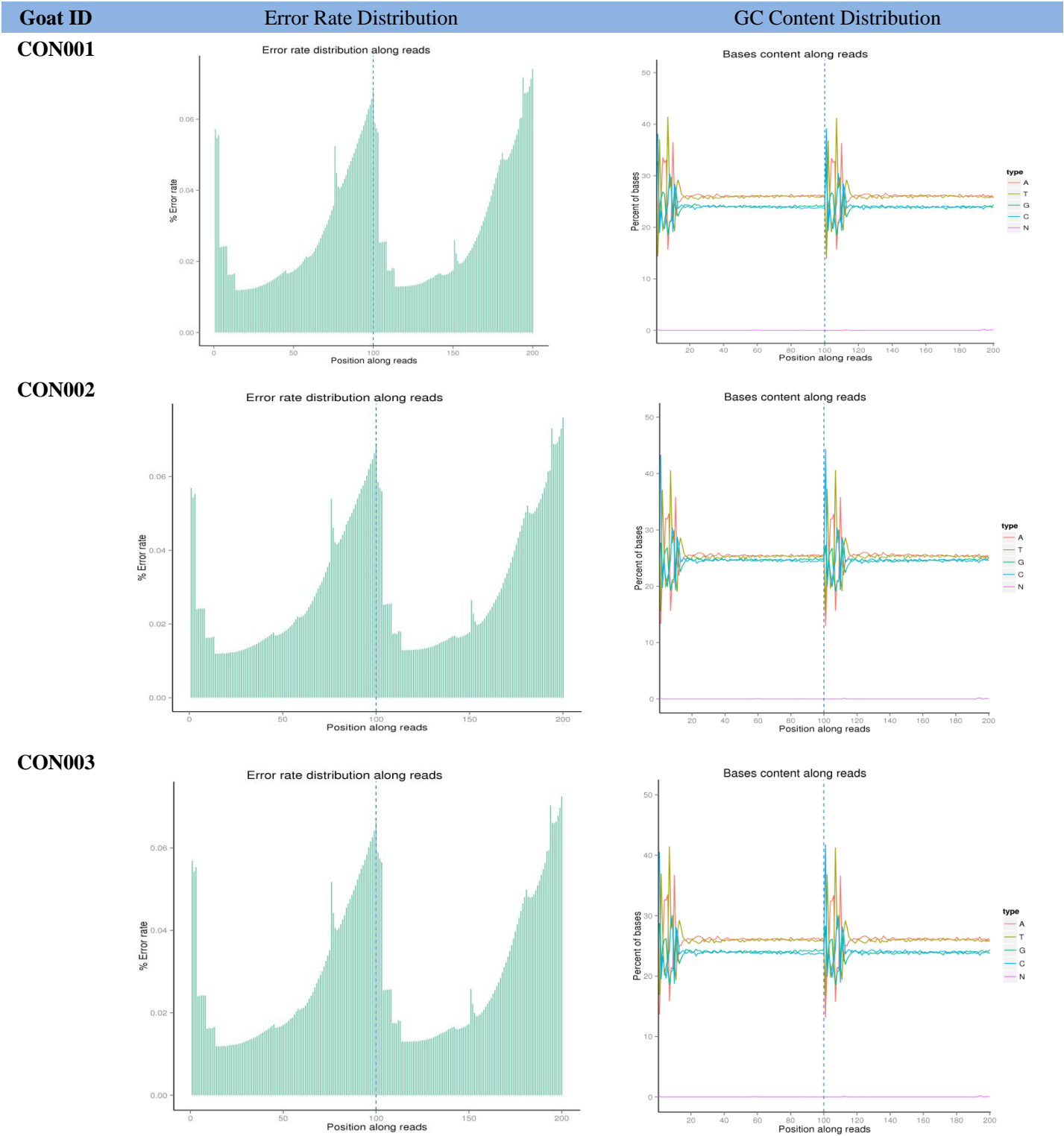

EA001

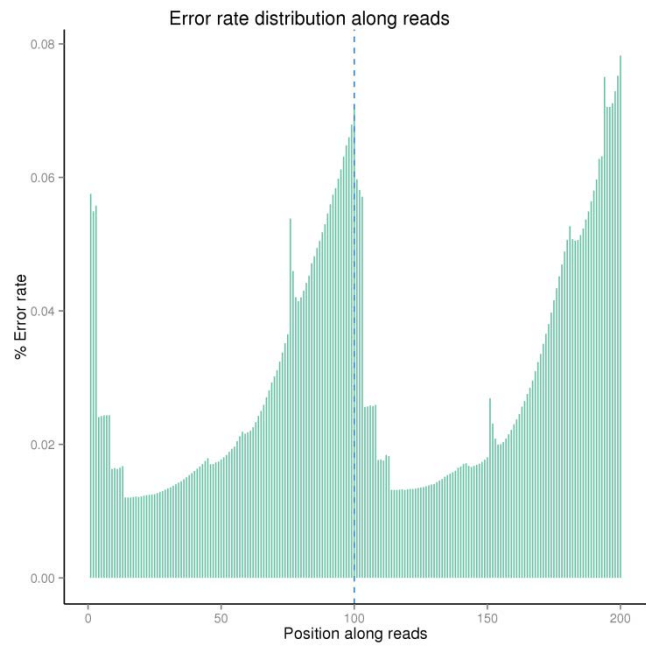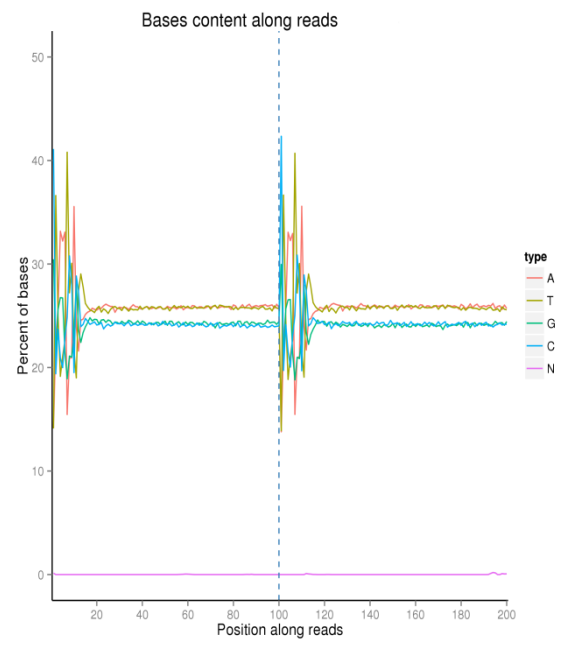

EA002

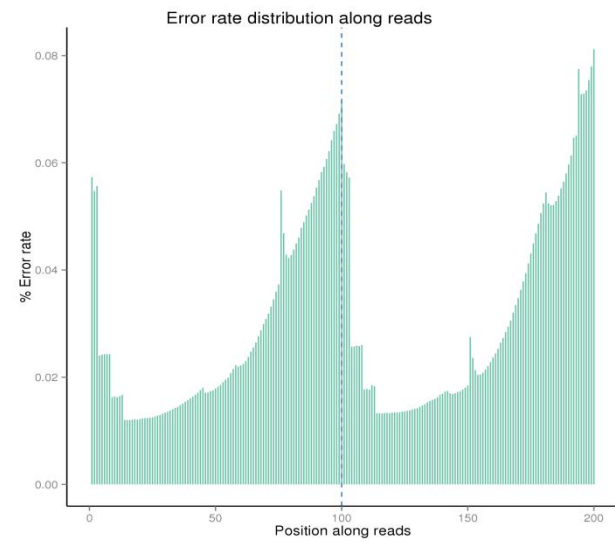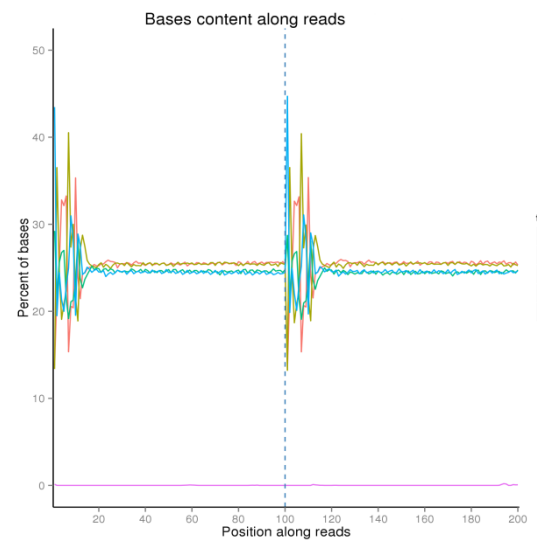

EA003

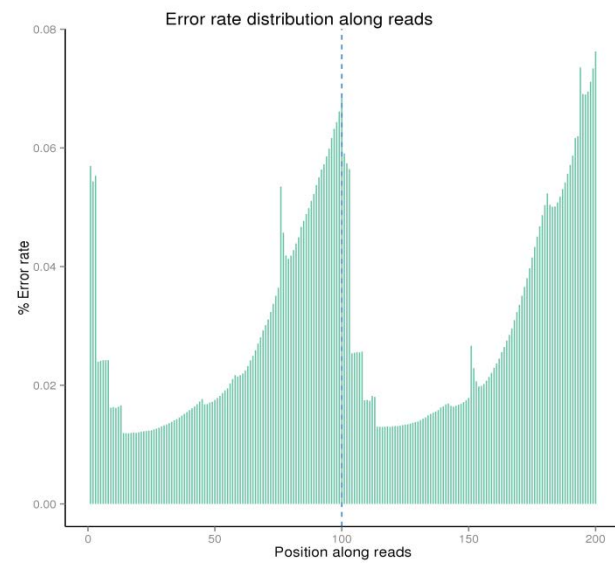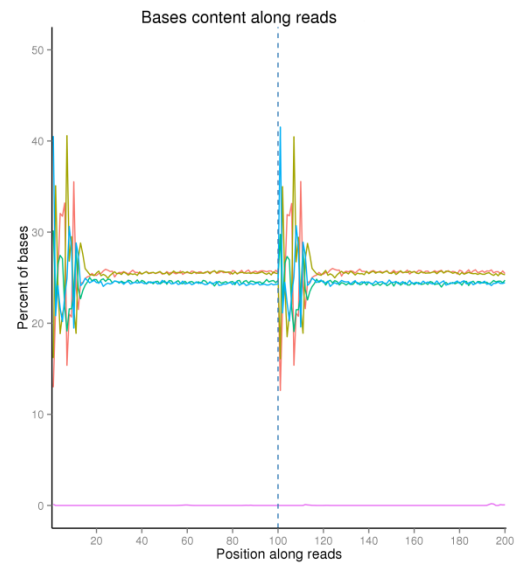

Supplement: Supplementary file 1 [file ijms-19-00002-s001.zip › figure S1.pdf]

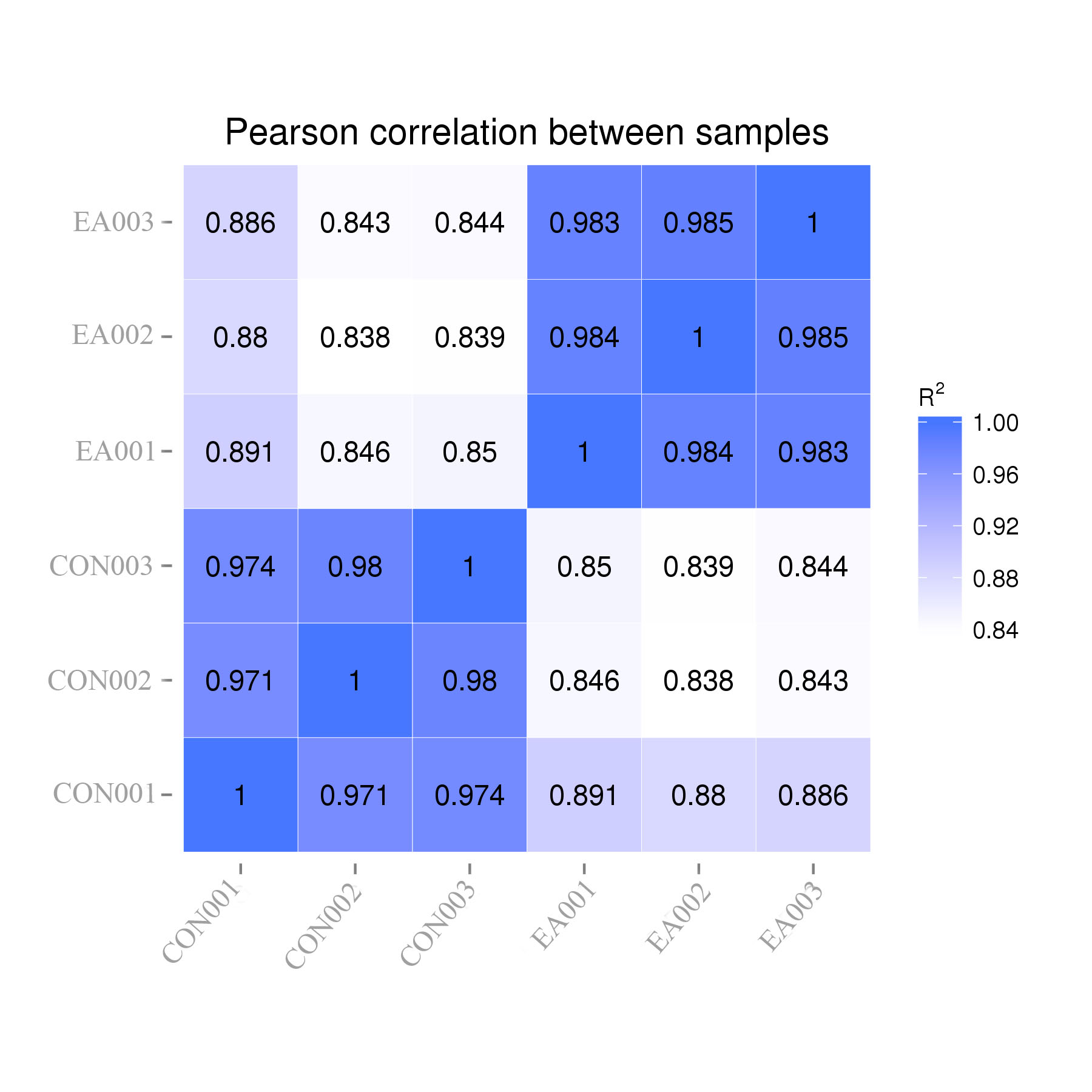

Supplement: Supplementary file 1 [file ijms-19-00002-s001.zip › Figure S2.jpg]
